# The comparative analyses of different diagnostic approaches in detection of gastroesophageal reflux disease in children

**DOI:** 10.1371/journal.pone.0187081

**Published:** 2017-11-02

**Authors:** Nina Ristic, Ivan Milovanovic, Milica Radusinovic, Marija Stevic, Milos Ristic, Maja Ristic, Darija Kisic Tepavcevic, Tamara Alempijevic

**Affiliations:** 1 Department of Gastroenterology, Hepatology and GI endoscopy, University Children’s Hospital, Belgrade, Serbia; 2 Department of Anesthesia, University Children’s Hospital, Belgrade, Serbia; 3 Emergency Surgery, Emergency Center, Clinical Centre of Serbia, Belgrade, Serbia; 4 Department for Nutrition and Food Safety Control, Institute for Public Health of Belgrade, Belgrade, Serbia; 5 Institute of Epidemiology, Belgrade Serbia; 6 Faculty of Medicine, University of Belgrade, Serbia; 7 Clinic for Gastroenterology and Hepatology, Clinical Center of Serbia, Belgrade, Serbia; Nationwide Children's Hospital Research Institute, UNITED STATES

## Abstract

**Objectives:**

The aim of this study was to compare the different diagnostic approaches in detection of gastroesophageal reflux disease in children presented with symptoms suggesting gastroesophageal reflux disease.

**Methods:**

The study design was cross sectional. The study retrospectively included all children who underwent combined multiple intraluminal impedance and pH (pH-MII) monitoring due to gastrointestinal and/or extraesophageal symptoms suggesting gastroesophageal reflux disease at University Children's Hospital in Belgrade, from July 2012 to July 2016.

**Results:**

A total of 218 (117 boys/101 girls), mean age 6.7 years (range 0.06–18.0 years), met the inclusion criteria. Gastroesophageal reflux disease was found in 128 of 218 children (57.4%) by pH-MII and in 76 (34.1%) children by pH metry alone. Using pH-MII monitoring as gold standard, sensitivity of pH-metry was lowest in infants (22.9%), with tendency to increase in older age groups (reaching 76.4% in children ≥ 9 years). The sensitivity of pH-metry alone in children with extraesophageal symptoms was 38.1%, while the sensitivity of pH-metry in children with gastrointestinal symptoms was 63.8%. Reflux esophagitis was identified in 31 (26.1%) of 119 children who underwent endoscopy. Logistic regression analysis showed that best predictors of endoscopic reflux esophagitis are the longest acid episode (OR = 1.52, p<0.05) and DeMeester reflux composite score (OR = 3.31, p<0.05). The significant cutoff values included DeMeester reflux composite score ≥ 29 (AUC 0.786, CI 0.695–0.877, p<0.01) and duration of longest acid reflux ≥ 18 minutes (AUC 0.784, CI 0.692–0.875, p<0.01).

**Conclusions:**

The results of our study suggested that compared with pH-metry alone, pH-MII had significantly higher detection rate of gastroesophageal reflux disease, especially in infants. Our findings also showed that pH-MII parameters correlated significantly with the endoscopically confirmed erosive esophagitis.

## Introduction

Gastroesophageal reflux (GER), retrograde flow of gastric contents into the esophagus, is a physiological phenomenon that occurs in healthy children several times a day after meals [1]. Gastroesophageal reflux disease (GERD) in pediatric patients is present when reflux of gastric contents is the cause of troublesome symptoms and/or complications [1,2]. Reflux esophagitis, represented with endoscopically visible breaks in the distal esophageal mucosa, is the most common consequence of esophageal injury caused by acid reflux [3].

Considering the fact that there is no "gold standard" for diagnosis of GERD, the establishing of well-defined criteria for its constitution is challenging and represent an area of intensive research in the field of pediatric gastroenterology. Furthermore, symptom-based diagnosis is not specific due to high prevalence of functional disorders and conditions that can mimic GERD, such as functional heartburn, cow's milk allergy and eosinophilic esophagitis [4–6]. It is clear that there is a urgent need to assess GERD in a more objective way, because the current clinical approach is subjected to free interpretation. Over-diagnosing of GERD resulted in over-prescription of acid-suppressing medications, even in infants [7].

Several studies have shown that combined multiple intraluminal impedance and pH (pH-MII) monitoring have much higher sensitivity of detection of reflux episodes than pH monitoring alone [8,9]. While pH sensitive electrode enables the differentiation between acid and non-acid reflux, MII monitoring discerns retrograde from anterograde bolus movement, identifies the composition of refluxate (liquid, gas and mixed liquid-gas) as well as the height reached by refluxate [10].

Despite wide use of pH-MII monitoring in children, there is still a lack of standard values for pediatric population. Moreover, studies assessing clinical and pH-MII predictors of reflux esophagitis are scarce [11,12]. Therefore, the aim of this study was to compare the results of different diagnostic methods, to investigate differences in clinical and pH–MII parameters between the different age groups and to determine predictors of reflux esophagitis in children presented with gastrointestinal (GI) and/or extraesophageal (EE) symptoms suggesting GERD.

## Materials and methods

### Study design

In the period between July 2012 and July 2016, all consecutive children up to 18 years old, who underwent pH-MII monitoring due to GI and/or EE symptoms suggestive of GERD at Department of gastroenterology, hepatology and GI endoscopy of the University Children's Hospital Belgrade were considered for enrollment in the study.

The inclusion criteria were as follows: (a) presence of GI symptoms suggestive of GERD including heartburn, epigastric pain, regurgitation/vomiting (in infants only if they are associated with failure to thrive and/or anemia) and/or back arching in infants; and/or (b) presence of EE symptoms suggestive of GERD including chronic cough, chronic hoarseness, recurrent pneumonia and/or apnea; and/or (c) presence of neurological disorders with GI and/or EE symptoms suggestive of GERD; and (d) children with GI symptoms and apnea, whose symptoms appeared at least 4 weeks before the pH-MII monitoring, and for other symptoms at least 8 weeks before monitoring. Exclusion criteria were: (a) proton pump inhibitors and/or H_2_ blockers usage during the week before the pH-MII monitoring, and (b) the presence of other diseases of the esophagus such as eosinophilic esophagitis and achalasia.

Children were stratified into three groups according to the age: (a) Group 1: children younger than 1 year (b) Group 2: children from 1 to 8 years old, (c) Group 3: children ≥ 9 years old.

All children who met the inclusion criteria and whose parent/caregiver gave written consent were included in the study. Of note, patients’ parents/caregivers were informed about the possibility that the obtained data might be used for the future research purposes. The study was approved by The Ethics Committee of University Children’s Hospital Belgrade (approval number 26/314).

### Combined pH-MII monitoring procedure

Multichannel intraluminal impedance-pH monitoring was performed by a pH-MII ambulatory system (*Sandhill Scientific*, *Denver*, *CO*). The pH-MII monitoring catheter system consists of an antimony pH sensor and 7 impedance electrodes representing 6 bipolar impedance channels. According to the length or height of patient, appropriate catheters (infant and pediatric) were used. Buffer solutions (pH 4.0 and 7.0) were used for pH electrode calibration before the procedure onset. The catheter was introduced nasally after at least 3 hours of fasting. For proper positioning of the catheter in infants up to 1 year of age, Strobel's formula has been used (length from the nostrils to lower esophageal sphincter in cm = 0.252 x length +5) [13]. X-ray was used to confirm placement of the pH probe so that the tip of the electrode lies over the third vertebral body above the diaphragm throughout the respiratory cycle [13]. Parents and children (if age-appropriate) were instructed to keep a careful diary both before and after catheter placement. Moreover, they were instructed to use an event marker to record times of meals, changes of body position, as well as symptoms and daily activities. The minimal duration of recording time was 20 hours. The pH-MII recordings were automatically analyzed using the software package (*AutoScan/ BioView Analysis*, *Sandhill Scientific*, *Highlands Ranch*, *CO*) and then reviewed manually. Recently introduced criteria on indications, methodology and interpretation of combined pH-MII monitoring in children were used for analyzing obtained impedance recordings [13].

Criteria for GERD based on results of pH monitoring was defined as reflux index (RI) (% proportion of time during which esophageal pH is below 4) > 10% in infants [8,12,14] and > 7% in older children [2]. A symptom was regarded as associated with reflux episode (RE) if it happened within 2 minutes after RE was detected by impedance. The association between RE detected by impedance and symptoms was estimated by calculation of the symptom index (SI), symptom sensitivity index (SSI) and symptom association probability (SAP).

Keeping in mind that there is no clear consensus, the MII results were defined as positive if the measurement fulfilled two out of three of the following criteria: SI ≥50%, SSI ≥10%, SAP ≥95% [8]. To avoid false-positive results, it was necessary to record at least 2 identical gastrointestinal or 5 identical extraesophageal symptoms (except for apnea whereas one episode was sufficient).

The following pH-MII parameters were analyzed as well: the number of reflux episodes (total, acidic, weakly acidic, non acidic), bolus elimination time, percentage of refluxate reaching proximal esophagus, DeMeester and Boix-Ochoa acid reflux composite score, number of children with symptoms during investigations, SI, SSI, SAP.

### Endoscopy

Data obtained from the children who underwent upper endoscopy were analyzed. Los Angeles (LA) classification for esophagitis was used for assessing the esophageal endoscopic findings and findings were considered positive if esophagitis grade A-D was detected.

### Statistical analysis

Normally distributed grouped data were expressed as mean with corresponding standard deviation, and compared using Student's t-test and analysis of variance (ANOVA). Nonparametric grouped data were expressed as median and compared with Mann-Whitney U test. Discrete variables were expressed as percentage, and for comparison between groups χ2 test and Fisher's exact probability test were used. A logistic regression model was used to determine the clinical predictors of GERD and pH-MII predictors of erosive esophagitis. Correlation was performed by Spearman correlation. Receiver operating characteristics (ROC) curve was used to assess the predictive power of various pH-MII variables. The ROC curves were calculated along with 95% confidence interval. Statistical significance was established at p <0.05. The SPSS 17.0 (*Chicago*, *IL*, *USA*) was used.

## Results

A total of 218 out of 243 children who underwent pH-MII monitoring met the inclusion criteria. The study population consisted of 117 (53.7%) boys and 101 (46.3%) girls, mean age 6.7±6.0 years (range 0.06–18.0 years). Isolated GI symptoms were present in 105 (48.2%), whereas isolated EE symptoms were present in 36 (16.5%) children. Concomitant presence of both, GI and EE symptoms, was present in 77 (35.3%) children. Types of symptoms and symptom distribution between different age groups are presented in Table 1.

**Table 1:** Symptom distribution between different age groups.

	Group 1 (n = 59)	Group 2 (n = 77)	Group 3 (n = 82)	
Isolated gastrointestinal symptoms	17 (28.8%)	36 (46.8%)	52 (63.4%)	p<0.001
Isolated extraesophageal symptoms	9 (15.3%)	14 (18.2%)	13 (15,9%)	
Concomitant appearance of gastrointestinal and extraesophageal symptoms	33 (55.9%)	27 (35.1%)	17 (20.7%)	
**Gastrointestinal symptoms**				
Vomiting/Regurgitation	49 (83%)	55 (71.4%)	34 (41.5%)	p<0.001
Heartburn	0	8 (10.4%)	35 (42.7%)	
Epigastric pain	1 (2%)	10 (13%)	27 (32.9%)	
Back arching	17 (28.8%)	6 (7.8%)	0	
**Respiratory symptoms**				
Cough	26 (44.1%)	38 (49.4%)	25 (30.4%)	p<0.001
Apnea	17 (28.8%)	1 (1.3%)	0	

The differences between age groups in all investigated pH-MII parameters, endoscopy findings and used treatment are presented in Table 2.

**Table 2:** Difference between different age groups in the results of pH, MII, endoscopy.

	Group 1 (n = 59)	Group 2 (n = 77)	Group 3 (n = 82)	p
Number of children with GERD determined by pH, n (%)	8 (13.6%)	26 (33.8%)	42 (51.2%)	<0.001**
Number of children with GERD determined by MII, n (%)	33 (55.9%)	19 (24.7%)	26 (31.7%)	<0.001^+^
Number of children with GERD determined by pH-MII, n (%)	35 (59.3%)	38 (49.4%)	55 (67.1%)	0.076
pH-metry				
Reflux episodes, mean (range)	16.3 (0–60)	30.9 (1–102)	52.4 (0–710)	<0.001*
Reflux index, %, mean (range)	4.6 (0–52.6)	9.2 (0–68.7)	15.9 (0.1–85.7)	<0.001*
DeMeester composite reflux score, mean (range)	15.8 (0.8–163.5)	30.5 (0.9–188.1)	51.4 (1.2–239.6)	<0.001*
Boix-Ochoa composite reflux score, mean (range)	15.3 (1–141.6)	29.1 (1.1–159.9)	46.8 (1.6–213.9)	<0.001*
Multiple intraluminal impedance				
Number of total reflux episodes, mean (range)	56.3 (1–125)	63.2 (5–197)	85.9 (12–246)	<0.001*
Number of acidic reflux episodes, mean (range)	24.2 (0–83)	46.4 (1–192)	69.4 (7–242)	<0.001**
Number of weakly acidic reflux episodes, mean (range)	30.9 (1–78)	16.3 (1–67)	15.5 (0–92)	<0.001^+^
Number of non-acidic reflux episodes, mean (range)	0.8 (0–12)	0.5 (0–8)	0.5 (0–16)	0.651
Bolus elimination time, s, mean (range)	2.2 (0–9)	3.2 (0.4–13.2)	5.1 (0.3–39.2)	<0.001*
% of proximal reflux episodes	57.3 (0–91.7)	47 (0–87.8)	52.3 (5.2–98)	0.020^#^
Number of children with symptoms recorded during impedance, n (%)	52 (88.1%)	53 (68.8%)	56 (68.3)	0.014^+^
SI %, mean (range)	42.5 (0–100)	38.2 (0–100)	46.4 (0–100)	0.340
SSI %, mean (range)	16.8 (0–68)	12.4 (0–80)	12.1 (0–85)	0.303
SAP %, mean (range)	69.7 (0–100)	61.6 (0–100)	65.1 (0–100)	0.604
Number of children with esophagitis/number of children in whom endoscopy was performed	3/19	7/44	21/56	0.027^&^
Proton pump inhibitor use after pH-MII (%)	9 (15.3%)	30 (39%)	44 (53.7%)	<0.001^+^

*Significant for group 1 vs. group 3 and group 2 vs. group 3.

**Significant for group 1 vs. group 2 and group 1 vs. group 3 and group 2 vs. group 3.

^+^Significant for group 1 vs. group 2 and group 1 vs. group 3.

^#^Significant for group 1 vs. group 2.

^&^Significant for group 2 vs. group 3.

SI- symptom index; SSI- symptom sensitivity index; SAP- symptom association probability.

GERD was determined by pH-MII in 128 (57.4%) children with symptoms suggestive of GERD, there was no statistical difference between different age groups (χ^2^ = 5.158, p>0. 05). Additionally, GERD was determined in 76 (34.1%) children by pH monitoring alone, and in 78 (35%) children by MII monitoring alone. When the pH-metry was compared to pH-MII, sensitivity of pH-metry was 59.4%. Particularly, sensitivity of pH-metry was very low in infants (sensitivity was 22.9%), with increasing tendency over older age groups (sensitivity was 68.4% in Group 2 and sensitivity was 76.4% in Group 3).

SI above 50% was found in 71 (32.5%) children overall, 28 (47.4%) infants in Group 1, 19 (24.7%) children in Group 2 and 24 (29.2%) children in Group 3. SSI above 10% was found in 65 (29.8%) children overall, 26 (44.1%) children in Group 1, 17 (22.1%) children in Group 2 and 22 (26.8%) children in Group 3 (data not shown). SAP above 95% was found in 78 (35.8%) children overall, 33 (55.9%) in Group 1, 19 (24.7%) children in Group 2 and 26 (31.7%) children in Group 3. All three parameters, as well as MII result (two positive out of three) were significantly higher in Group 1 compared to Group 2 and 3 (p<0.01) (data not shown). Sensitivity of MII alone was 60.2%, with the highest sensitivity of 94.3% in the youngest age group, whereas sensitivity was 50% and 47.3% in Group 2 and 3, respectively.

The sensitivity of pH-metry alone in children with EE symptoms was 38.1%, while sensitivity of pH-metry in children with GI and both GI and EE was 63.8% and 61.9%, respectively.

Difference between age groups in the number of reflux episodes (total, acidic, weakly acidic and non-acidic) showed significantly higher number of total and acidic reflux episode in older children, as well as significantly higher number of weakly acidic RE in infants (Table 2). Moreover, infants had significantly more reflux episodes that reached proximal esophagus compared to other groups. The most proximal extent of impedance positive reflux was 15 cm in children overall (12 cm in Group 1, 14 cm in Group 2 and 15 cm in Group 3) (data not shown). The mean proximal extent in Group 1 was 10.7±1.5 cm, in Group 2 11.5±0.74 cm, and in Group 3 12.5±1.7 cm, there was significant statistical difference between groups (ANOVA, F = 26.199, p<0.001) (data not shown).

Very high correlation between DeMeester and Boix-Ochoa score was found (r = 0.989, p<0.01, 95% CI).

Differences in pH-MII results among children with confirmed esophagitis and those with normal endoscopy finding are presented in Table 3.

**Table 3:** Differences between children with esophagitis and children with normal endoscopy.

	Children with esophagitis (n = 31)	Children without esophagitis (n = 88)	p
Age, mean (range)	11.0 (0.25–18)	7.11 (0.17–18)	<0.001
Gender (male/female)	19/12	47/41	0.448
pH-metry alone			
Reflux episodes, mean (range)	43.7 (0–101)	35.8 (1–114)	0.198
Reflux index, %, mean (range)	29.9 (0–80.5)	11.7 (0–85.7)	<0.001
Multiple intraluminal impedance			
Number of total reflux episodes, mean (range)	91.6 (1–197)	78.0 (5–246)	0.193
Number of acidic reflux episodes, mean (range)	78.0 (0–192)	57.2 (1–242)	0.036
Number of weakly acidic reflux episodes, mean (range)	13.3 (0–92)	19.6 (0–79)	0.105
Number of non-acidic reflux episodes, mean (range)	0.2 (0–2)	0.8 (0–16)	0.198
Bolus elimination time, s, mean (range)	6.1 (0–39.2)	3.8 (0.3–18)	0.023
% of proximal reflux episodes	45.7 (0–84.6)	52.7 (0–100)	0.313
DeMeester composite reflux score	95.5 (0.8–239.6)	37.9 (0.8–235.7)	<0.001
Boix-Ochoa composite reflux score	83.8 (1–187.4)	36.0 (1–213.9)	<0.001
SI %, mean (range)	59.2 (0–100)	50.6 (0–100)	0.170
SSI % mean (range)	6.2 (0–22)	12.1 (0–85)	0.034
SAP % mean (range)	67.3 (0–100)	70.1 (0–100)	0.695
Number of children with GERD determined by pH, n (%)	25 (80.6%)	37 (42%)	<0.001
Number of children with GERD determined by MII, n (%)	8 (25.8%)	32 (36.4%)	0.378
Number of children with GERD determined by pH-MII, n (%)	27 (87.1%)	55 (62.5%)	0.013

SI- symptom index; SSI- symptom sensitivity index; SAP- symptom association probability.

Logistic regression failed to determine potential predictors of GERD in terms of age, gender and the type of symptoms (p>0.05).

When pH-MII results were compared by type of symptoms, a significant difference was found only in symptom sensitivity index (SSI). SSI was 15.6% (range 0–85%) in children with EE symptoms, whereas in children with GI and both GI and EE symptoms, SSI was 7.6% (range 0–77%) and 12.1% (range 0–68%), respectively. Moreover, this index was statistically significantly higher in group of children with EE symptoms compared to the other subgroups (p<0.01).

Endoscopy was performed in 119 (54.6%) study participants, and consequently identified reflux esophagitis in 31 (26.1%) children. Reflux esophagitis was the most prevalent (38.5%) in Group 3, while its prevalence was 22.6% and 15.8% in the Group 2 and 1 respectively. Sensitivity of endoscopy was 32.9% and specificity 89.2%.

Reflux esophagitis significantly correlated with reflux index (ρ = 0.429, p<0.01), duration of the longest acid episode (ρ = 0.247, p<0.01), the total number of acidic reflux episodes (ρ = 0.251, p<0.01), the total number of weakly acidic reflux episodes (ρ = 0.22, p<0.05), all bolus exposure percent time (ρ = 0.215, p<0.05), acid bolus exposure percent time (ρ = 0.251, p<0.01) and DeMeester acid reflux composite score (ρ = 0.435, p<0.01).

Logistic regression analysis of pH-MII variables showed that the best predictors of endoscopic reflux esophagitis are duration of the longest acid episode (OR = 1.52, p<0.05) and DeMeester acid reflux composite score (OR = 3.31, p<0.05).

**Fig 1:**
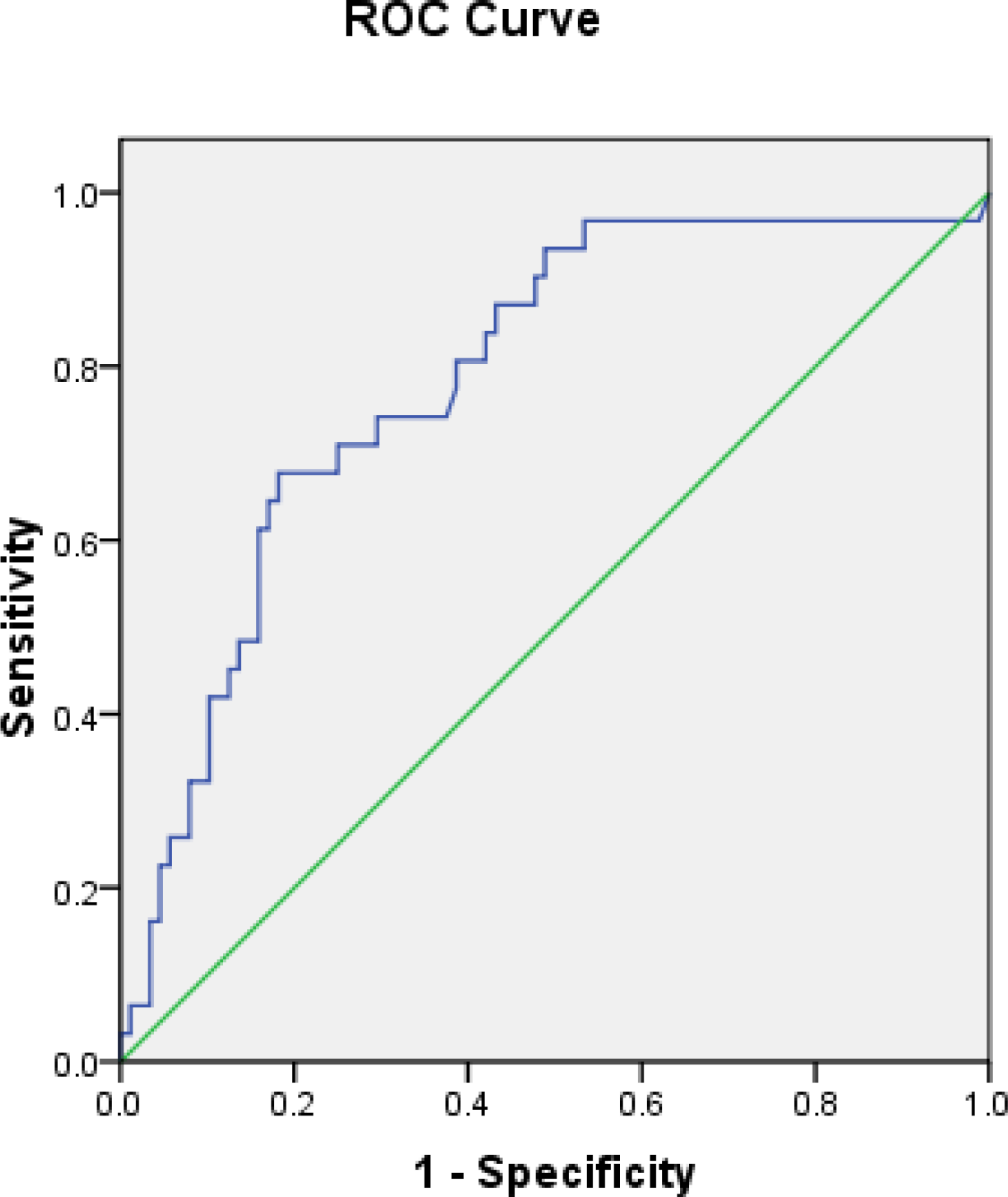
Receiver operating characteristic (ROC) curve for the value of DeMeester score to predict erosive esophagitis (cutoff ≥ 29, AUC 0.786, p<0.01).

**Fig 2:**
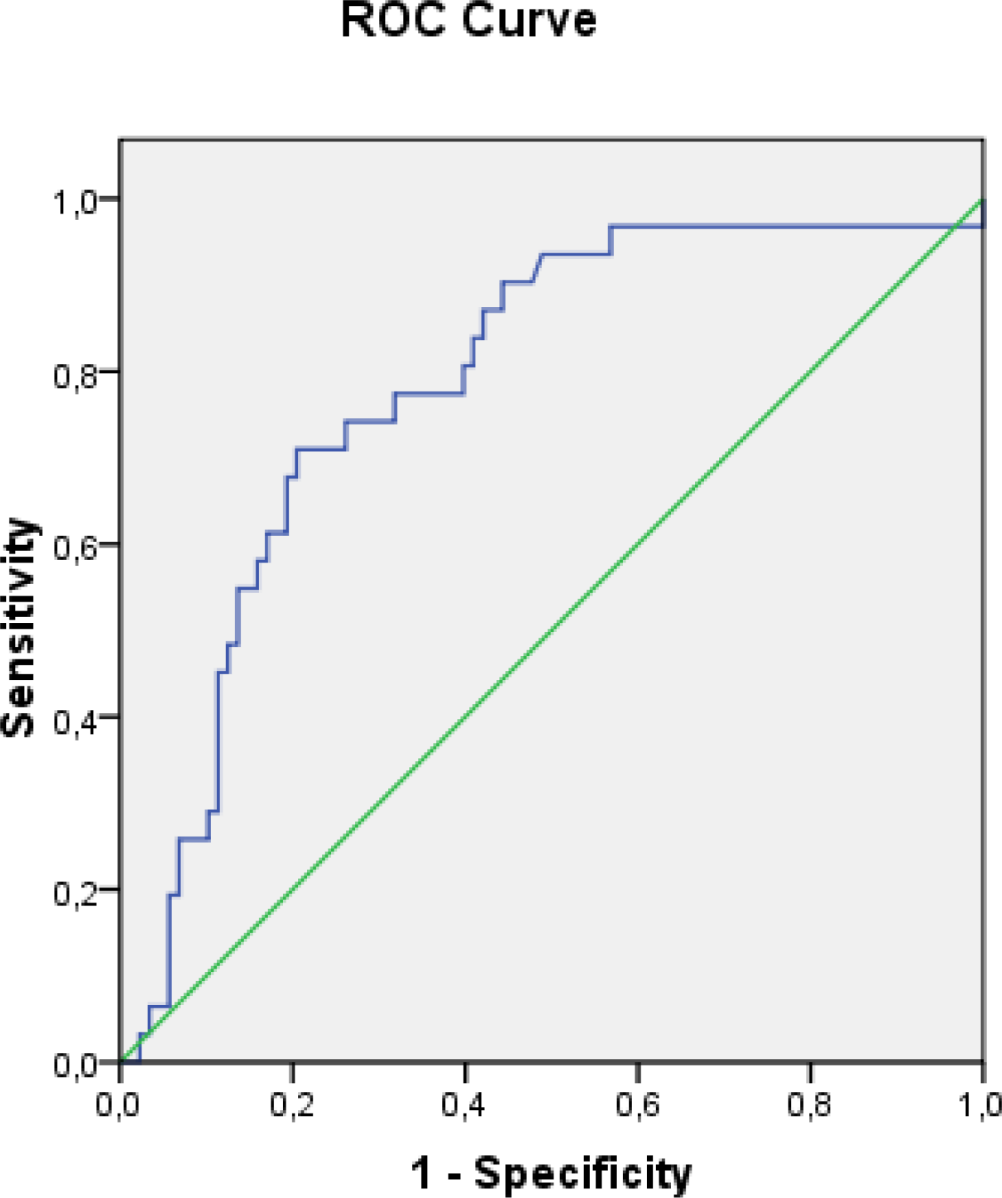
Receiver operating characteristic (ROC) curve for the duration of the longest acid reflux to predict erosive esophagitis (cutoff ≥ 18, AUC 0.784, p<0.01).

ROC analyses yielded the significant cutoff values of pH-MII variables, including DeMeester acid reflux composite score ≥ 29 (AUC 0.786, CI 0.695–0.877, p<0.01) (Fig 1) and duration of the longest acid reflux ≥ 18 minutes (AUC 0.784, CI 0.692–0.875, p<0.01) (Fig 2).

Seventeen children (14 boys/3 girls) with neurological disorders were enrolled in the study, median age 1.77 (range 2 months-14.7 years). Positive results on pH-MII monitoring were found in 10 (58.8%) children. Six (35.3%) children were positive on pH monitoring. Children with neurological disorders experienced statistically significantly higher number of reflux episodes that reached proximal esophagus (t = 4.114, p<0.01) compared to other children, whereas significant differences in other pH-MII parameters were not found.

Among studied population 19 infants were presented with apnea and GI symptoms suggesting gastroesophageal reflux disease. Although pH-MII results were positive in 12 (63.2%) infants (12 infants were MII positive, and only 2 pH positive), we did not found symptom association between apneas and reflux episodes.

## Discussion

One of the problems is that pH-MII monitoring in children still lacks standard values for pediatric population. Furthermore, there is no clear consensus concerning abnormal MII findings. Some studies showed disagreement between symptom-reflux association analysis parameters in pediatric gastroesophageal reflux disease investigations [15]. The German Pediatric Impedance group defined MII study as abnormal if the measurement fulfilled the following criteria: SI ≥50% or a high number of reflux episodes (>70 episodes in patients aged 1 year or older and >100 episodes in infants) [9]. Other authors defined positive symptom association when both SI and SSI are positive or when SAP is positive [16]. We decided to use symptom indexes as reference measures and two out of three positive criteria for defining MII abnormality [8].

The three widely used pH-metry scores in children are DeMeester score, Boix-Ochoa score and Johnson-DeMeester score. As considered until recently, the Boix-Ochoa score is the most accurate score to be used in pediatrics for the diagnosis of GERD [17]. The recent diagnostic accuracy study showed that all three above mentioned scores have high sensitivity and specificity of the pH-metry measurements, whereas Johnson-DeMeester score has a higher risk of false-negative results [18]. The same study showed very high correlation between DeMeester and Boix-Ochoa score (r = 0.978, p<0.01, 95% CI) which is in concordance with our results. These results implicate that both scores can be equally used in pH studies.

Our results highlight the fact that the addition of MII to conventional pH monitoring significantly increases sensitivity of the test in infants and children with suspected GERD. This study showed that the sensitivity of pH-metry alone is especially low in infants, and that it increases with age. Moreover, our findings indicate that 40% of infants and children with an abnormal finding on pH-MII would not be identified by pH-metry alone. Up to 77% infants compared to 24% children above 8 years of age would be undiagnosed with pH-metry alone. The sensitivity of pH-metry (using pH-MII monitoring as gold standard) in children with isolated EE symptoms is 38.1%, whereas in children with GI symptoms with or without concomitant EE symptoms it is almost 2-fold higher. However, even in the group with GI symptoms more than 35% of children with an abnormal finding on pH-MII would not be diagnosed by pH-metry alone. These findings are in concordance with previous reports [8,9]. Higher sensitivity of pH-metry in children above 8 years, which was found in our study, could be partially explained by possible presence of selection bias due to inclusion of children with more troublesome symptoms and frequent use of proton pump inhibitor test in this age group. Keeping in mind the fact that in our sample more than one-third of children above 8 years with GI symptoms suspected for GERD were pH-MII negative, it is important to emphasize the high prevalence of functional dyspepsia in this age group, as previously reported [8]. Furthermore, this implicated low diagnostic value of GI symptoms in differential diagnosis between GERD and functional dyspepsia, even in older children, which is in agreement with *Hojsak et al* [8]. However, it is important to mention that reporting of symptoms is unreliable in children under the age of approximately 8 years [1]. The reliability is even lower in infants and neurologically impaired children who are unable to report “troublesome” symptoms and in whom symptoms (or signs) reporting depends on their parents/caregivers.

In our study, symptom association between apneas and reflux episodes was not found. From the published studies, it is evident that in the majority of infants with apnea or apparent life-threatening event, GER is not the cause. However, in Joint recommendation of NASPGHAN and ESPGHAN from 2009 was stated that in rare occasions in which a relation between symptoms and GER is suspected or in those with recurrent symptoms, MII/pH monitoring in combination with polysomnographic recording and precise, synchronous symptom recording may aid in establishing potential causal relationship [2]. In the same recommendations apnea spells are included in signs that may be associated with gastroesophageal reflux [2]. A relation between GER and short, physiologic apnea has been shown [19]. One recently published study demonstrated that pathologic apnea can occur as a consequence of GER [20].

In contrast with some previous studies [8], our data showed higher number of total and acidic reflux episodes in older children. However, we found greater number of weakly acidic episodes in infants, as in previous reports [21]. The prevalence of weakly acidic reflux episodes varies considerably by age, from about 60% in infants to less than 30% in children. These data clearly demonstrate the differences in age-related chemical composition of refluxate [21]. Our data showed significantly more reflux episodes that reached proximal esophagus in infants, which is in concordance with previous findings [8,21]. Interestingly, comparing pH-MII results of children with and without neurological disorders, the only significant difference was in percent of proximal reflux episodes, which was higher in children with neurological disorders. In this group of patients extraesophageal manifestations are more prevalent (especially cough), supporting hypothesis that a direct stimulation of airway structures is involved in the genesis of RE-associated cough [22].

The prevalence of erosive esophagitis in children varies, from 12.4% reported in multicenter study from 2008 [23] to 34.6% found in a single center study from 2001 [24]. The problem is that patients who had endoscopy were not patients with GERD symptoms only, therefore the prevalence of esophagitis in children might be underestimated [25]. It has been well recognized that endoscopy has high specificity (90%-95%) for GERD [26]. However, a poor sensitivity of around 50% has been reported [27]. In our study, using pH-MII monitoring as gold standard, we found low sensitivity of endoscopy (32.9%), which is in agreement with results from previous studies [8,11].

Several studies investigated possible correlation between endoscopic findings and clinical symptoms in this vulnerable cohort of children and failed to demonstrate any association [12,28,29]. Most of them failed to confirm correlation between reflux episodes detected by MII monitoring and endoscopy findings [12,29]. Study by *Hojsak et al*. showed that children with GI symptoms and endoscopically proven esophagitis had a higher number of all reflux episodes detected by pH-MII, but not by pH-metry alone [8]. The other survey established the relationship between the parameters of pH-MII and the presence of endoscopic reflux esophagitis in children [11]. The same study set up the cutoff values of DeMeester score ≥ 21, the duration of longest acid reflux ≥ 17 minutes, and the occurrence of acid reflux for more than 5 minutes to predict the presence of reflux esophagitis [11]. In concordance with the latter, our study showed that predictors of reflux esophagitis are DeMeester acid reflux composite score ≥ 29 and duration of the longest acid episode ≥ 18 minutes. These findings suggest that macroscopic mucosal changes of the esophagus are more common in patients with increased acid exposure, as was previously reported in studies in adults [30]. However, this does not rule out the possibility that weakly acidic and non-acidic refluxate affects mucosal integrity of esophagus. It has been shown that lower baseline impedance is associated with esophagitis ≥ LA grade B and may be caused by longer acid, as well as by longer bolus exposure [12]. Further investigations are needed in order to elucidate these hypotheses.

This study had several limitations dominantly related to its retrospective nature and the lack of healthy control group due to ethical reasons. Although we enrolled all consecutive patients with suspected GERD referred to our hospital, we could not completely eliminate selection bias. No validated parent- or patient- reported gastroesophageal reflux questionnaires have been used prior to monitoring. Both questionnaires for infants (Infant Gastroesophageal Reflux Questionnaire (I-GERQ) [31] and Infant Gastroesophageal Reflux Questionnaire Revised (I-GERQ-R) [4]) as well as 5-item questionnaire for children 7 to 16 years of age [32] have been shown to be valid and reliable for documentation and monitoring of reported symptoms. All questionnaires were validated using abnormal pH probe studies and/or abnormal esophageal biopsies as gold standards, and none by using abnormal pH-MII monitoring. Moreover, it has been suggested that the questionnaires are more useful for follow-up of patients than for diagnosing GERD [33]. Furthermore, endoscopy was not performed in all patients from our study population. The proportion of patients in whom endoscopy was performed is especially low in infants, in whom the prevalence of erosive esophagitis is generally considered to be low. In addition to that, endoscopy rate is lower in children with normal pH-MII results (if pH-MII preceded endoscopy). One of the limitations is that histology hasn’t been performed in all patients who underwent endoscopy. This is partly due to the known fact that the correlation between the endoscopic features of GERD and histology is poor, especially in cases of nonerosive disease [34]. On the other hand, excellent correlation between endoscopy findings and histology in patients with erosive reflux disease lessens the importance of histopathology in these patients [35]. Some authors consider histology as non-mandatory and others recommend biopsies only to rule out other pathology [31]. Due to cross-sectional design of the study we did not provide information on the follow-up of patients and treatment effects.

## Conclusions

The results of our study suggested that compared with pH-metry alone, pH-MII had significantly higher detection rate of GERD in all age groups, and especially in infants. Our findings also showed that pH-MII parameters correlated significantly with the endoscopically confirmed erosive esophagitis. Even though, endoscopy is still the diagnostic mode of choice for the confirmation of mucosal lesions, pH-MII parameters could be considered as potential markers for estimation of integrity of esophageal mucosa, which should be further investigated. Although, standardization is mandatory, pH-MII monitoring shows promising performances for the diagnosis of GERD, and tendency to become the gold standard for detection of this disorder in pediatric population.
